# *Vital Signs:* Containment of Novel Multidrug-Resistant Organisms and Resistance Mechanisms — United States, 2006–2017

**DOI:** 10.15585/mmwr.mm6713e1

**Published:** 2018-04-06

**Authors:** Kate Russell Woodworth, Maroya Spalding Walters, Lindsey M. Weiner, Jonathan Edwards, Allison C. Brown, Jennifer Y. Huang, Sarah Malik, Rachel B. Slayton, Prabasaj Paul, Catherine Capers, Marion A. Kainer, Nancy Wilde, Alicia Shugart, Garrett Mahon, Alexander J. Kallen, Jean Patel, L. Clifford McDonald, Arjun Srinivasan, Michael Craig, Denise M. Cardo

**Affiliations:** ^1^Division of Healthcare Quality Promotion, National Center for Emerging and Zoonotic Diseases, CDC; ^2^Tennessee Department of Health; ^3^Iowa Department of Public Health.

## Abstract

**Background:**

Approaches to controlling emerging antibiotic resistance in health care settings have evolved over time. When resistance to broad-spectrum antimicrobials mediated by extended-spectrum β-lactamases (ESBLs) arose in the 1980s, targeted interventions to slow spread were not widely promoted. However, when Enterobacteriaceae with carbapenemases that confer resistance to carbapenem antibiotics emerged, directed control efforts were recommended. These distinct approaches could have resulted in differences in spread of these two pathogens. CDC evaluated these possible changes along with initial findings of an enhanced antibiotic resistance detection and control strategy that builds on interventions developed to control carbapenem resistance.

**Methods:**

Infection data from the National Healthcare Safety Network from 2006–2015 were analyzed to calculate changes in the annual proportion of selected pathogens that were nonsusceptible to extended-spectrum cephalosporins (ESBL phenotype) or resistant to carbapenems (carbapenem-resistant Enterobacteriaceae [CRE]). Testing results for CRE and carbapenem-resistant *Pseudomonas aeruginosa* (CRPA) are also reported.

**Results:**

The percentage of ESBL phenotype Enterobacteriaceae decreased by 2% per year (risk ratio [RR] = 0.98, p<0.001); by comparison, the CRE percentage decreased by 15% per year (RR = 0.85, p<0.01). From January to September 2017, carbapenemase testing was performed for 4,442 CRE and 1,334 CRPA isolates; 32% and 1.9%, respectively, were carbapenemase producers. In response, 1,489 screening tests were performed to identify asymptomatic carriers; 171 (11%) were positive.

**Conclusions:**

The proportion of Enterobacteriaceae infections that were CRE remained lower and decreased more over time than the proportion that were ESBL phenotype. This difference might be explained by the more directed control efforts implemented to slow transmission of CRE than those applied for ESBL-producing strains. Increased detection and aggressive early response to emerging antibiotic resistance threats have the potential to slow further spread.

*On April 3, 2018, this report was posted online as an *MMWR *Early Release on the *MMWR* website (https://www.cdc.gov/mmwr).*

## Introduction

The emergence and spread of antibiotic resistance threatens to outpace the development of new antimicrobials, and slowing the spread of these organisms has become a priority. Among Enterobacteriaceae, the family of pathogens most frequently associated with health care–associated infections (*1*), resistance to the broad-spectrum antimicrobials extended-spectrum cephalosporins and carbapenems has been driven largely by the spread of plasmid-mediated resistance genes encoding extended-spectrum β-lactamases (ESBLs) and carbapenemases, respectively. In the United States, ESBL-producing Enterobacteriaceae were first reported in 1988 (*2*). The emergence of these ESBL-producing isolates limited the options available for treatment, but these organisms remained susceptible to some first-line therapies, including carbapenems. In general, facilities independently selected approaches to control spread, which often included core infection control practices, such as hand hygiene, and placing patients with ESBL-producing strains in single rooms under Contact Precautions.

Enterobacteriaceae resistance to even broader spectrum antimicrobials, including carbapenems, was reported with increasing frequency beginning in 2001 (*3*). Rapid spread of these carbapenem-resistant Enterobacteriaceae (CRE) in parts of the United States and other countries (*4*,*5*) highlighted a need to more aggressively control CRE transmission. In 2009, CDC created CRE-specific guidance, which was endorsed by the Healthcare Infection Control Practices Advisory Committee (*6*). This guidance included recommendations for additional interventions when CRE was identified at a health care facility, including laboratory surveillance of clinical cultures and targeted patient screening to identify health care contacts with asymptomatic colonization. This CRE-specific guidance was updated in 2013 and 2015 (https://www.cdc.gov/hai/organisms/cre/cre-toolkit/index.html) and was highlighted by CDC in a 2013 report (*7*).

In 2017, CDC outlined a new effort to react rapidly to novel multidrug-resistant organisms (*8*); this approach includes encouraging health care facilities and public health authorities to respond to single isolates of an emerging antibiotic-resistant pathogen. The strategy rests on these five pillars: 1) rapid detection of targeted pathogens and their resistance mechanisms, 2) on-site infection control assessments by trained experts to identify gaps in infection prevention, 3) screening of exposed contacts to identify asymptomatic colonization, 4) coordination of the response among facilities, and 5) continuing these interventions until transmission is controlled. Detection and control efforts can extend from the index facility to other facilities that share patients.

To support this approach, CDC established the Antibiotic Resistance Laboratory Network (ARLN) (https://www.cdc.gov/drugresistance/solutions-initiative/ar-lab-networks.html) to improve national capacity to rapidly detect and respond to antibiotic resistance. ARLN provides carbapenemase testing for two emerging antibiotic resistant pathogens, CRE and carbapenem-resistant *Pseudomonas aeruginosa* (CRPA), at 56 state and local public health laboratories and screening for asymptomatic CRE and CRPA carriage at seven regional laboratories (*9*). Carbapenemase-producing strains were targeted for detection and response in part because of their previously demonstrated propensity for spread. CDC also expanded funding to state and local health departments to increase capacity and build expertise in responding to these and other emerging antibiotic resistance threats.

For this report, data from a national health care–associated infections surveillance system were reviewed to determine if the more directed approach applied for CRE was associated with differences in the percentage of Enterobacteriacae health care–associated infections that were CRE compared with those that had the ESBL phenotype. In addition, findings from the first 9 months of the enhanced response to emerging resistant organisms are described.

## Methods

**Percentage of Enterobacteriaceae with CRE or ESBL phenotypes in the National Healthcare Safety Network, 2006–2015.** Included in the analysis were central line–associated bloodstream infections (CLABSIs) and catheter-associated urinary tract infections (CAUTIs) associated with *Escherichia coli* or *Klebsiella pneumoniae* and reported to CDC’s National Healthcare Safety Network (NHSN) during 2006–2015 from adult medical, surgical, or medical/surgical intensive care units at short-stay acute care hospitals. The Centers for Medicare & Medicaid Services’ (CMS) Hospital Inpatient Quality Reporting Program mandated reporting of CLABSI and CAUTI data to NHSN starting in 2011 and 2012, respectively; data from previous years represent voluntary reporting or reporting to comply with state or local mandates. National pooled mean percentages for Enterobacteriaceae with CRE phenotype (isolates resistant to imipenem, meropenem, doripenem, or ertapenem), and ESBL phenotype (isolates that tested intermediate or susceptible to carbapenems and intermediate or resistant to ceftazidime, cefepime, ceftriaxone, or cefotaxime) were calculated. Log binomial regression models were used to estimate the average annual change in the proportion of *E. coli* and *K. pneumoniae* that had a CRE or ESBL phenotype. P-values <0.05 were considered statistically significant. Sensitivity analyses were performed to account for the change in hospitals reporting to NHSN each year. The results of the log binomial regression model were confirmed by a robust variance Poisson model.

**Enhanced detection and response.** CRE and CRPA (*P. aeruginosa* resistant to imipenem, meropenem, or doripenem) isolates were submitted to ARLN laboratories for testing for carbapenemases. Among Enterobacteriaceae, *E. coli, K. oxytoca, K. pneumoniae*, and *Enterobacter* spp. were targeted for submission. Testing at ARLN laboratories included carbapenemase production testing and molecular detection of genes encoding for the five carbapenemases of primary public health concern: *Klebsiella pneumoniae* carbapenemase (KPC), New Delhi metallo-beta-lactamase (NDM), Verona integron encoded metallo-beta-lactamase (VIM), imipenemase (IMP), and oxacillinase-48-like carbapenemase (OXA-48). ARLN laboratories were asked to report positive findings to local public health authorities and CDC within 1 day and to submit testing summaries to CDC monthly.

For each carbapenemase-producing isolate detected, CDC guidance recommends that state health department staff members contact the health care facility to review infection control measures and consider performing on-site infection control assessments. If indicated, contacts of the index patient are screened to detect transmission; testing capacity for this screening is provided through ARLN. Response activities continue until transmission is controlled. Screening results were stratified by whether the screening took place in a short-stay acute care hospital or a post–acute care facility (i.e., long-term acute care hospital or nursing home).

## Results

**FIGURE 1:**
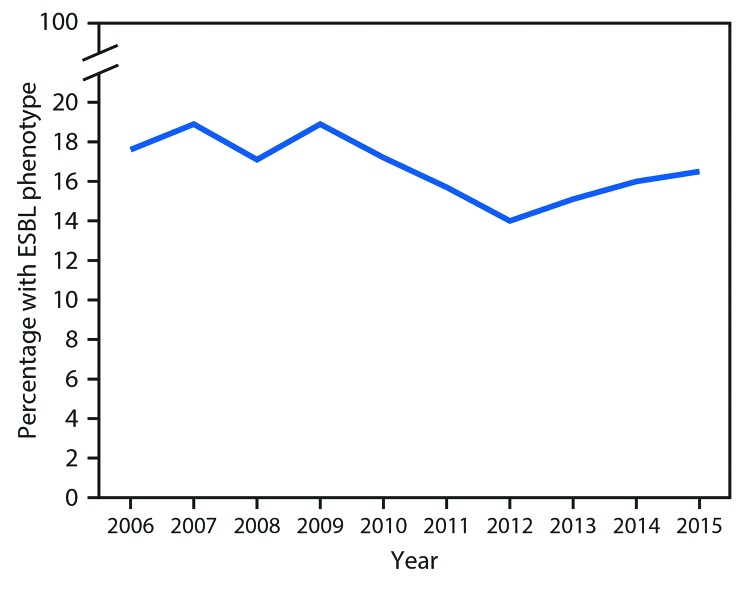
Percentage of *Escherichia coli* and *Klebsiella pneumoniae* isolates from selected health care–associated infections* with the extended-spectrum-β-lactamase (ESBL) phenotype reported as nonsusceptible to extended-spectrum cephalosporins^†^ — National Healthcare Safety Network, United States, 2006–2015 * Central line–associated bloodstream infections and catheter-associated urinary tract infections. ^^†^^ Nonsusceptible to at least one extended-spectrum cephalosporin.

**FIGURE 2:**
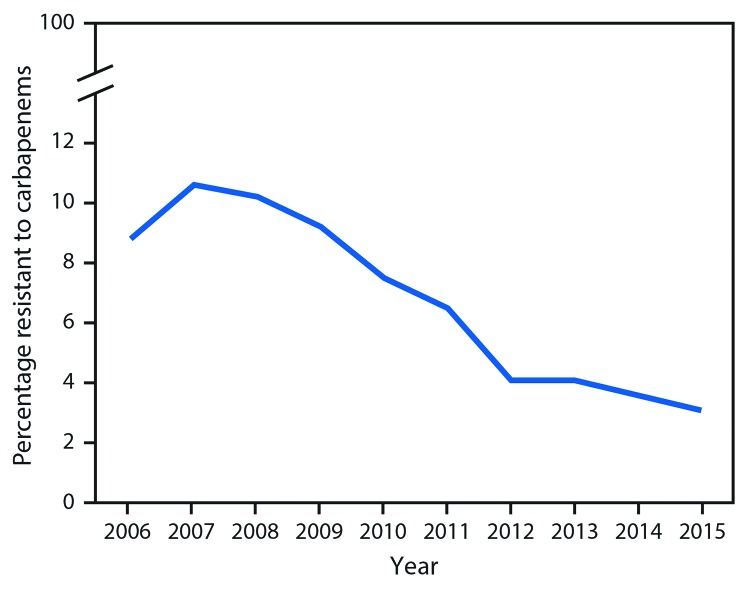
Percentage of *Escherichia coli* and *Klebsiella pneumoniae* isolates from selected health care–associated infections* reported as resistant to a carbapenem — National Healthcare Safety Network, United States, 2006–2015 * Central line–associated bloodstream infections and catheter-associated urinary tract infections.

**Percentage of Enterobacteriaceae with CRE or ESBL phenotypes in the National Healthcare Safety Network, 2006–2015.** Among short-stay acute care hospitals, the percentage of *Klebsiella* and *E. coli* isolates with the ESBL phenotype remained relatively stable, ranging from 17.6% (116 of 659 isolates) in 2006 to 16.5% (694 of 4,211) in 2015, with a peak of 18.9% in 2009 (Figure 1). The percentage of CRE declined from 8.8% (35 of 397 isolates) in 2006 and 10.6% (64 of 604) in 2007 to 3.1% (115 of 3,718) in 2015 (Figure 2). During 2006–2015, the annual percentage of isolates with the ESBL phenotype declined an average of 2% (RR = 0.98, p = 0.009); during the same period, the proportion that were CRE decreased 15% per year (RR = 0.85, p<0.001). Results were unchanged when the analysis was limited to facilities that reported in all years.

**Enhanced detection of and response to carbapenemase-producing organisms.** During the first 9 months of 2017, among 4,442 CRE and 1,334 CRPA isolates that were tested for carbapenemases from 32 states, 1,401 (32%) CRE and 25 (1.9%) CRPA were carbapenemase producers (Table 1). Among the carbapenemase-producing isolates, 221 (15.5%) expressed carbapenemases other than KPC. Of isolates tested, 1,422 (25%) were collected in the first quarter of 2017, 2,141 (37%) in the second quarter, and 2,213 (38%) in the third quarter. During this period, the median time from specimen collection to CDC notification decreased from 37 to 13 days. The percentage of carbapenemase-producing isolates varied by organism and was highest among *Klebsiella* species (65%). Among carbapenemase-producing CRE, the most commonly identified carbapenemase was KPC (1,232 of 1,401 isolates, 88%); VIM was the most common carbapenemase identified in CRPA (18 of 25, 72%) (Table 1).

**TABLE 1:** Carbapenemase testing, by organism — Antibiotic Resistance Laboratory Network laboratories and CDC laboratory, specimens collected January 1–September 30, 2017

Organism	Total		KPC		NDM		OXA-48		VIM		IMP	
	Tested* no.	Positive^†^ no. (%)	Tested no.	Positive no. (%)	Tested no.	Positive no. (%)	Tested no.	Positive no. (%)	Tested no.	Positive no. (%)	Tested no.	Positive no. (%)
**Total**	**5,776**	**1,426 (25)**	5,755	1,234 (21)	5,570	134 (2.4)	5,323	65 (1.2)	4,724	30 (0.6)	4,068	16 (0.4)
**Enterobacteriaceae**	**4,442**	**1,401^§^ (32)**	4,430	1,232 (28)	4,247	134 (3.2)	4,050	65 (1.6)	3,448	12 (0.3)	2,827	11 (0.4)
*Klebsiella *spp.	**1,439**	**942 (65)**	1,437	862 (60)	1,359	74 (5.4)	1,295	42 (3.2)	1114	4 (0.4)	744	1 (0.1)
*E. coli*	**789**	**144 (18)**	783	83 (11)	755	43 (5.7)	719	20 (2.8)	665	0 (0)	585	0 (0)
*Enterobacter *spp.	**1,538**	**201 (13)**	1,537	194 (13)	1,468	14 (1.0)	1,387	0 (0)	1,201	0 (0)	1,063	3 (0.3)
Other	**346**	**72 (21)**	345	53 (15)	336	3 (0.9)	322	2 (0.6)	256	7 (2.7)	238	7 (2.9)
Unspecified	**330**	**42 (13)**	328	40 (12)	329	0 (0)	327	1 (0.3)	212	1 (0.5)	197	0 (0)
***Pseudomonas aeruginosa***	**1,334**	**25^§^ (1.9)**	1,325	2 (0.2)	1,323	0 (0)	1,273	0 (0.0)	1,276	18 (1.4)	1,241	5 (0.4)

**Abbreviations:** IMP = imipenemase; KPC = *Klebsiella pneumoniae* carbapenemase; NDM = New Delhi metallo-beta-lactamase; OXA-48 = oxacillinase-48-like carbapenemase; VIM = Verona integron encoded metallo-beta-lactamase.

* Number of isolates tested.

^†^ Positive for at least one of the five carbapenemases tested (IMP, KPC, NDM, OXA-48, or VIM).

^§^ 53 isolates were positive for more than one mechanism tested (28 KPC and NDM; 24 NDM and OXA-48; one KPC and VIM).

To identify asymptomatically colonized health care contacts of index patients, 1,489 screening tests for carbapenemases were performed during 70 surveys (defined as all screening tests performed at a single facility within a 14-day period) in 50 facilities. A median of 10.5 contacts (interquartile range = 2–25) were screened per survey. Overall, 11% of screening tests were positive for at least one of the five carbapenemases of primary public health concern (Table 2). A higher percentage of post–acute care facility contacts screened positive for carbapenemases (14% [147 of 1,074 contacts]) than did contacts from short-stay acute care hospitals (5.8% [21 of 365]) (p<0.01). Screening tests performed increased from 363 in the first quarter of 2017, to 732 in the third.

**TABLE 2:** Screening tests for carbapenem-resistant Enterobacteriaceae colonization, by facility type — Antibiotic Resistance Laboratory Network laboratories and CDC laboratory, specimens collected January 1ؘ–September 30, 2017

Carbapenemase	Total*		Post–acute care facility^†^		Short-stay acute care hospital	
	Screened^§^ no.	Positive no. (%)	Screened no.	Positive no. (%)	Screened no.	Positive no. (%)
**Total**	**1,489**	**171^¶^ (11)**	1,074	147 (14)	365	21 (5.8)
KPC	**1,480**	**122 (8.2)**	1,065	103 (10)	365	16 (4.4)
NDM	**1,480**	**6 (0.4)**	1,065	6 (0.6)	365	0 (0)
OXA-48	**1,311**	**0 (0)**	896	0 (0)	365	0 (0)
VIM	**1,488**	**34 (2.3)**	1,073	30 (2.8)	365	4 (1.1)
IMP	**1,311**	**9 (0.7)**	896	8 (0.9)	365	1 (0.3)

**Abbreviations:** IMP = imipenemase; KPC = *Klebsiella pneumoniae* carbapenemase; NDM = New Delhi metallo-beta-lactamase; OXA-48 = oxacillinase-48-like carbapenemase; VIM = Verona integron encoded metallo-beta-lactamase.

* Includes 50 screening tests without a reported facility type, three of which were positive for KPC.

^†^ Includes long-term acute care facilities, skilled nursing facilities, and inpatient rehabilitation facilities.

^§^ Number screened refers to individual screening tests. Not all screening swabs were tested for all five mechanisms.

^¶^ Seven tests were positive for more than one mechanism tested (four KPC and NDM, and three KPC and VIM).

**Illustrative examples.** Public health responses using this new approach have identified single cases without transmission, transmission within facilities, and spread to multiple facilities. Examples from two states are presented to illustrate these efforts.

In October 2017, the Tennessee Department of Health contacted CDC regarding identification of an NDM and OXA-48–producing *Klebsiella pneumoniae* isolate through ARLN. Infection control assessment and screening of hospital contacts was completed and results returned within 48 hours of identification of carbapenemase presence. No transmission was identified. Because the index patient had a recent health care exposure in another country, ARLN regional laboratories expanded their services to perform CDC-recommended admission screening for patients with a history of overnight health care stays outside the United States during the preceding 6 months (*10*).

In April 2017, the Iowa Department of Public Health contacted CDC regarding IMP identified in a *Proteus* species isolated from a nursing home resident. The state health department assessed infection control practices and performed a point prevalence survey that identified five additional colonized residents among 30 surveyed at the nursing home. The health department conducted additional infection control assessments to ensure adherence to recommended practices and two follow-up surveys of the nursing home wing, which did not identify any additional cases.

## Conclusions and Comments

Although the proportion of *Klebsiella* spp. and *E. coli* that had either an ESBL or CRE phenotype both declined during 2006–2015, larger decreases and a lower overall percent resistant were observed for the CRE phenotype. This difference might be attributable, at least in part, to the more directed response employed to slow the spread of CRE once it was identified. Although CDC’s containment approach had not yet been fully initiated when the decline in CRE began, these data suggest that an early aggressive response, as outlined in CRE-specific infection prevention recommendations released beginning in 2009 (*6*), can slow emergence and even decrease the occurrence of infections from resistant pathogens. As laboratory capacity improved, ARLN testing volume and public health responses increased over the first three quarters of 2017, demonstrating that recent investments in detection and response capacity are facilitating prompt identification of and response to emerging resistant organisms. Notably, 221 isolates with non-KPC carbapenemases were identified; these rare forms of resistance have the potential to add to the U.S. CRE burden and represent an important opportunity to prevent the spread of novel resistance at its earliest stage. Findings from these enhanced prevention efforts are being used to further refine detection and prevention strategies.

Contact screening identified previously undetected transmission and appeared to have the highest yield in post–acute care facilities with higher acuity patients. Challenges in these settings that might facilitate transmission of resistant organisms include long duration of facility stay, less aggressive use of transmission-based precautions because of concerns about resident quality of life, high staff turnover rates, and less expertise and training in infection control. Previous work has also identified these settings as potential amplifiers of CRE transmission (*11*), underscoring the importance of providing ongoing support to these facilities when targeted resistant organisms are identified. This support includes infection control assessments to improve adherence to recommended interventions and screening of contacts to identify asymptomatic carriers.

Although this analysis focused on carbapenemase-producing organisms, the containment strategy can prevent the spread of other emerging antimicrobial resistant pathogens, including *Candida auris* and pan-resistant bacteria. Using existing surveillance systems, including ARLN, further work is under way to better identify and understand new threats, including those that are emerging outside the United States. CDC continues to work to develop tests for new resistance mechanisms that can be made available via ARLN. Resistance is constantly evolving, and the containment strategy and ARLN are designed to be flexible and nimble to rapidly detect and respond to new threats.

Despite improvements in capacity to detect carbapenemases in clinical isolates and asymptomatic carriers through ARLN, challenges remain. Transmission in one facility in a region has the potential to affect all of the facilities and patients in a region through patient sharing; therefore, recognition by health care facilities of the importance of an aggressive, early, and coordinated response is needed to ensure responses are timely and comprehensive. Mathematic modeling of the containment strategy based on a single U.S. state’s patient transfer network suggests that an intervention resulting in a 20% reduction in transmission would result in approximately 1,600 fewer clinical cases, a relative reduction of about 76%, 3 years after introduction (CDC, unpublished data, 2018). In addition, commitment from health care personnel and health care facilities to improve adherence to infection control interventions that can prevent transmission, especially in post–acute care settings, is necessary to prevent amplification of emerging resistance. For situations in which a targeted form of antimicrobial resistance has emerged more widely in a region, containment strategies might be less effective; additional work is required for these situations to identify the optimal strategies to reduce the prevalence of endemic resistant organisms. Finally, current interventions are challenging to implement and sustain; new interventions to reduce transmission are needed to supplement currently available prevention measures.

Public health departments, because of their expertise and ability to work across health care facilities, are uniquely positioned to facilitate these responses to emerging antimicrobial resistance. Since 2009, CDC has provided resources to develop state and local health care–associated infection programs; currently, CDC supports approximately 500 persons in state and local health departments to work on health care-associated infections and antimicrobial resistance. Details on funding provided to each state to combat antimicrobial resistance are provided in CDC’s antimicrobial resistance map (https://wwwn.cdc.gov/arinvestments).

The findings in this report are subject to at least four limitations. First, resistance data in NHSN are collected using the final interpretations of resistant, intermediate, or sensitive, and this analysis does not account for differences among laboratories in the breakpoints used for interpretation or for changes in breakpoints over time. Enterobacteriaceae breakpoints for carbapenems and some cephalosporins were lowered during the analysis period. This might have resulted in an increase in isolates reported as resistant in later years of this analysis and could have resulted in an underestimation of any reductions in CRE or ESBLs described. Second, NHSN data analyzed for this report represent only isolates from two infection types (CAUTI and CLABSI); changes in colonization or other infection types would not be identified. Third, although greater reductions were seen in the percentage of organisms that were CRE compared to those with the ESBL phenotype, this analysis is unable to identify the exact cause for this difference. Finally, some states and health care facilities with colonization testing capacity chose to perform screening in-house rather than through the ARLN regional laboratory; these tests are not reported to ARLN and therefore are not included in this report, resulting in an underestimation of the true volume of screening conducted.

Limiting the spread of emerging forms of antibiotic resistance is a public health priority, and a timely and coordinated effort among health care facilities, local and state health departments, and CDC is needed to accomplish this goal. Research is already under way to expand control strategies through innovative approaches such as patient decolonization and microbiome manipulation, along with a focus on identifying strategies to decrease the time from specimen collection to public health response. Fortunately, with the parallel development of an enhanced prevention strategy for emerging antimicrobial resistance and implementation of advanced laboratory testing in ARLN, the critical tools for controlling the spread of antimicrobial resistance are now available nationwide. In the first year of ARLN implementation, CDC and state and local public health departments and public health laboratory partners have effectively increased the capacity to identify and respond to high concern organisms to prevent transmission of resistant pathogens. Although some challenges remain, this national public health strategy represents a critical step in the effort to decrease the impact of resistant pathogens.

**Key Points**

• The emergence and spread of antibiotic resistance threatens to outpace the development of new antibiotics. Slowing the spread of emerging resistance is a CDC priority to protect persons and help slow the development of antibiotic resistance overall.

• Infection data from the National Healthcare Safety Network from 2006-2015 were analyzed to calculate changes in the annual proportion of selected pathogens that were nonsusceptible to extended-spectrum cephalosporins (ESBL phenotype) or resistant to carbapenems (carbapenem-resistant Enterobacteriaceae [CRE]).

• The percentage of ESBL phenotype Enterobacteriaceae decreased by 2% per year; by comparison, the CRE percentage decreased by 15% per year.

• The proportion of Enterobacteriaceae infections that were CRE remained lower and decreased more over time than the proportion that were ESBL phenotype. This difference might be explained by the more directed control efforts implemented to slow transmission of CRE than those applied for ESBL-producing strains.

• These data suggest that an early aggressive response, as outlined in CRE-specific infection prevention recommendations released beginning in 2009, can slow emergence and even decrease the occurrence of infections from resistant pathogens.

• In 2017, CDC outlined a new effort to react rapidly to novel multidrug-resistant organisms; this approach includes encouraging health care facilities and public health authorities to respond to even single isolates of an emerging antibiotic-resistant pathogen.

• From January to September 2017, carbapenemase testing was performed by the Antibiotic Resistance Lab Network for 4,442 CRE and 1,334 carbapenem-resistant *Pseudomonas aeruginosa* (CRPA) isolates; 32% and 1.9%, respectively were carbapenemase-producers. Among the carbapenemase-producing isolates, 221 (15.5%) expressed carbapenemases other than *Klebsiella pneumoniae* carbapenemase. Carbapenemases can make germs resistant to some of our most powerful drugs, carbapenems.

• Additional information is available at https://www.cdc.gov/vitalsigns/.
